# Editorial: Designing Carrier-Free Immobilized Enzymes for Biocatalysis

**DOI:** 10.3389/fbioe.2022.924743

**Published:** 2022-06-22

**Authors:** Susana Velasco-Lozano, Javier Rocha-Martin, José C. S. dos Santos

**Affiliations:** ^1^ Heterogeneous Biocatalysis Laboratory, CIC BiomaGUNE, San Sebastián, Spain; ^2^ Department of Biochemistry and Molecular Biology, Faculty of Biology, Complutense University of Madrid, Madrid, Spain; ^3^ Instituto de Engenharias e Desenvolvimento Sustentável, Universidade da Integração Internacional da Lusofonia Afro-Brasileira, Campus das Auroras, Redenção, Brazil

**Keywords:** enzyme immobilization, carrier-free immobilization, biotransformation, multi-enzymes, biocatalysis

Global challenges in energy, resources, environment and sustainable chemical processing are leading us to minimize the use of natural resources, toxic materials, energy, and the generation of waste and pollutants. Enzymatic biocatalysis development evolves to strengthen the ongoing required process improvements of current industrial chemical transformations, where enzymes are naturally available, efficient biocatalysts exhibiting high specificity and enantioselectivity. However, for their industrial application, enzymes must possess high stability under large-scale producing conditions (such as high substrate and product concentrations, temperature, presence of solvents), where enzymes easily become inactive due to their low stability under such unnatural conditions or the almost null activity toward non-natural substrates. In this context, enzyme immobilization is a strategy that can facilitate their stabilization, purification, and reuse in the industry (Cavalcante et al., 2021; da S. Moreira et al., 2021; Reis et al., 2019). Enzymes can be immobilized on water-insoluble supports or without supports. Among carrier-free immobilization methods, the Cross-Linked Enzyme Aggregates (CLEA) emerged as a promising technology capable of manufacturing robust immobilized protein aggregates. In general, the preparation of CLEA involves the first stage of precipitation of enzymatic molecules followed by a cross-linking step managed by the action of a bifunctional reagent added to the reaction system, the cross-linker. As a result, the aggregates become insoluble, maintaining high catalytic activity, excellent stability, and low production cost.

Another possibility of immobilizing enzymes free of support is the cross-linked enzyme crystals (CLEC). Compared to soluble enzymes, CLEC is more robust, controllable in size, resistant to organic solvents, and inactivates by heat or proteolysis. However, in contrast with CLEA, CLEC requires purified enzymes to achieve the required enzyme crystals formation, thus hampering their implementation at significant scale processes.

CLEAs’s physical and catalytic properties are strongly influenced and controlled through the preparation process (Table 1). Several works have summarized the most reported variables leading to robust CLEA biocatalysts from a wide variety of different enzymes, such as hydrolases, oxidoreductases, lyases, transferases, and isomerases for different industrial applications (Talekar et al., 2013; Cui and Jia, 2015; Velasco-Lozano et al., 2016; Sheldon, 2019; Velasco-Lozano, 2020). During the first step, yielding protein aggregates, the precipitation conditions, and precipitating agents may lead enzyme aggregates exhibiting different catalytic properties. This stage must ensure enzymes’ insolubilization maintains an active or even improved conformation. The second step involves the irreversible maintenance of the formed aggregates by their cross-linking, thus the molar ratio cross-linker to enzyme, the time and the conditions will trigger CLEA exhibiting different catalytic properties. This stage, must yield enough cross-linking degree to preserve together with the enzyme aggregates under no precipitation conditions but at the same time must leave enough enzyme flexibility to preserve enzyme activity. Furthermore, during both preparation stages, incorporating various additives such as protein feeders, polymers, detergents, magnetic particles, polysaccharides and so on allows the enhancement of enzyme immobilization yield, enzyme recovered activity, and enzyme substrate specificity and improved biocatalyst stability and reusability. Nevertheless, there is no unique approach or conditions where all enzymes achieve their immobilization as CLEA biocatalysts. Thus for each enzyme or enzyme combination, the optimization of each variable involved in the preparation process must be conducted before yielding a highly robust CLEA.

**TABLE 1:** Parameters governing CLEA’s main properties.

Preparation step	Variables	Possible affected properties
Enzyme preparation	• Enzyme and total protein concentration	
	• Enzymes ratio when working with more than one enzyme	• Activity
	• Additive incorporation (detergents, protein feeders, magnetic particles, polymers, polysaccharides, etc)	• Immobilization yield
	• Enzyme bioimprinting	• Size (particle size, pore-size)
	• Enzyme’s reactive surface residues	• Morphology (rigidity, softness, resistance to shearing stress)
Precipitation	• Precipitant nature		• Stability (thermal, pH, storage and operational)
	• Precipitant concentration		
	• Precipitation time	• Stabilization of multimeric enzymes
	• Stirring speed	• Reusability (easy handling and recovery, avoid clump formation)
	• Temperature	
	• pH	• Selectivity
Cross-linking	• Cross-linker nature and length	
	• Molar ratio cross-linker to enzyme
	• Cross-linking conditions (time, temperature, pH, etc)
	• Stirring speed

CLEA has been successfully employed for a vast diversity of biotechnological applications, from preparing pharmaceuticals (as sem-synthetic penicillins and cephalosporins), nutraceuticals, cosmetic ingredients, etc carbohydrate conversion processes, among others (Sheldon et al., 2013; Sheldon, 2019). More recently, CLEAs have been applied in the biomass conversion, aimed at valorizing food by-products, where feruloyl esterase CLEAs have been successfully employed to obtain high-valued bioactive compounds as phenolic acids from cereals and grains wastes (Grajales-Hernández et al., 2021); and to revalorize dairy by-products producing lactofructose syrup from whey surplus (Wilson et al., 2022). Additionally, the CLEA approach has enabled the stabilization of complex multi-domain enzymes whose applications at large-scale preparation processes are hampered by their low stability *in vitro* conditions (Wagner et al., 2021).

Furthermore, multi-task robust aggregates can be prepared by co-precipitation and cross-linking of two or more enzymes in “combined CLEAs” (combi-CLEA). These can catalyze multiple cascade reactions with different potential applications; as to simplify multi-step processes in one-pot (as the combi-CLEA of β-Galactosidase and glucose isomerase synthesizing lacto-fructose syrup (Wilson et al., 2022)); for the efficient cofactor regeneration (as the combi-CLEA formed by glycerol dehydrogenase and NADH oxidase (Xu et al., 2020) and the combi-CLEA of BVMO and FDH for the synthesis of *S*-omeprazole); and the hydrolysis of complex molecules requiring more than one enzymatic activity (as the combi-CLEA of xylanase and mannanase employed in the hydrolysis of lignocellulose waste for the production of second generation of biodiesel (Bhattacharya and Pletschke, 2015)); among others.

Beyond random enzyme-aggregates formation, other recent approaches include lectin-mediated enzyme aggregates employing Concavalin A to agglutinate glycosylated proteins as GOx and HRP enzymes prior aggregate’s cross-linking (Zhang et al., 2016). These aggregates facilitate the uptake of substrates and the colocalization of cascade enzymes with nanoscale proximity (6.9 nm), thus enabling a faster consumption of the intermediate and attaining more than five times a decrease in Km and 1.5 times higher specificity constant.

The intelligent CLEA or magnetic CLEAs (mCLEA) technology is gaining prominence in industrial applications since the separation of the immobilized enzyme from other solids’ reaction systems is facilitated. mCLEA can be quickly and separated and recovered magnetically on an industrial scale using standard commercial equipment, enabling the combination of biocatalysis and downstream processing. Mainly, m-CLEAs are highly useful when separating them from a mixture of solids, as in the case of slurry processes. Industrial application of mCLEAs and m-combi-CLEAs has the possibility of using them in the complex conversions of polysaccharides such as starch (Torabizadeh and Montazeri, 2020), lignocellulose for the production of first- and second-generation biofuels (Cruz-Izquierdo et al., 2015), for the synthesis of bioactive molecules as aglycone (Wei et al., 2022), and for clarification and flavor enhancers in food and beverages (Dal Magro et al., 2018). Likewise, magnetic CLEAs can be used in the conversion of biomass as in the valorization of keratin-rich poultry industry wastes where keratinase m-CLEA showed enhanced activity and stability than the soluble enzyme (Lotfi et al., 2021), as well as in the enantioselective reduction of prochiral ketones catalyzed by magnetic combi-CLEAs of his-tagged ketoreductase and his-tagged glucose oxidase (Peschke et al., 2019), for dye-degradation in wastewater (Wang et al., 2021).

New CLEAs for industrial applications have gained prominence in this context since they generate correct environmental products and sustainable chemical systems. In the context of enzymatic immobilization, the work presented (Cavalcanti et al.) demonstrated that entrapment in alginate beads is a promising technique for the immobilization of Tannase from *A. fumigatus* CAS21. With this strategy, the thermal and pH stabilities of the tannase were considerably improved for the Calginate derivative. The catalytic potential of the prepared tannase was efficient for large-scale biotechnological applications. The authors proved that the Ca-alginate preparation containing tannase is a suitable biocatalyst used in a packed bed reactor (PBR) to treat tannery effluents for successive operational cycles in PBR (Cavalcanti et al.). The study developed by the authors suggests that the treatment of effluents from the leather industry in PBR with a Ca-alginate derivative is an alternative for the reduction of tannins and phenolic compounds and the clarification of effluents. This was proven in the study since, after enzymatic treatment, the effluent showed improved and suitable characteristics to increase the efficiency of subsequent treatment steps, such as biological treatment (Cavalcanti et al.).

A study on the thermodynamic properties of microencapsulation of enzymes in liposomes was presented in our Research Topic by Alonso-Estrada et al. According to the authors, the encapsulation technique in colloidal and vesicular carriers allows enzymes for different applications, such as the mycolytic enzymes used to control phytopathogenic fungi.

In this way, the different systems free from solid carriers are examples of new methods for treatments with enzymes, drugs, toxins, and antimicrobials, which suggest strong links with their applications in medicine, agriculture, and livestock. Second (Alonso-Estrada et al.) are becoming hot topics in new treatments. The enzymes chitinase and laminarinase have an affinity for soy lecithin liposomes. Research advances on the thermodynamic properties of microencapsulation of enzymes in liposomes will be considered for process optimization in future studies and uses. In the presented study, the stability of enzyme preparations was increased, and their antifungal properties (Alonso-Estrada et al.).

Considering the researchers’ study published in our Research Topic (Chauhan et al.), the selected approach to immobilization may differ from enzyme to enzyme, carrier to carrier. For different use, mainly relying on the particularities of each unique process, the standards for measuring the robustness of the immobilized enzyme remain the same (Chauhan et al.). Agreeing with these authors, commercially applied immobilized enzymes need to be relatively active, relatively selective (to decrease cross-reactions), relatively stable (to decrease value through means of efficient reuse), value-intensive (contribution of low value, therefore economically viable), safe to use (to satisfy protection regulations), and innovative. However, the productivity of almost all immobilized enzymes is relatively lower than that of chemical processes. This is due to diffusion restrictions for researchers (Chauhan et al.). Activity retention for porous carriers is regularly below 50% at most of the enzymatic load in a biocatalytic reaction system. In line with the Research Topic, the enhancement of carrier-less enzymes, including CLEA or CLEC, may defer the use of the non-catalytic mass provider, the intrinsic disadvantages related to carriers’ immobilized enzymes. The authors consider that carrier less biocatalytic systems appear to be very attractive, as no scaffolding/matrix is required, no modification or matrix activation is required, little leaching effect is observed, and the complete absence of aldehyde cross-linking chemicals from the benefits of such biocatalytic systems. The authors Chauhan et al. suggest that in progressive research in the area, the future seems to be bright in creating advanced techniques to immobilize different enzymes, which would increase the efficiency of the enzyme by many times (Chauhan et al.).

In the work of Blanco-Llamero et al. CLEAs and combi-CLEAs were produced and characterized from Alcalase®, Viscozyme®, and Celluclast® from commercial enzymatic solutions for biomass pretreatment. The results published by the authors Blanco-Llamero et al., as relevant results, the CLEAs of the three enzymes were successfully produced with high immobilization rates (above 80%). Furthermore, the immobilization results were followed up with the results of the stability tests that were achieved by incubating the biocatalysts under different conditions and by the results of proof-of-concept reactions in microalgae biomass. It was observed that the best conditions obtained showed their usefulness in the pre-treatment of microalgae, facilitating cell disruption and reducing possible variations due to the instability of soluble enzymes, as was achieved by increasing the extraction yield and the structural morphology of the cell wall by SEM (Blanco-Llamero et al.). In conclusion, the authors suggest the application of CLEAs as a promising technology for the pretreatment of microalgae to improve the stability of enzymes commonly used in this biomass and decrease the number of solvents and energy used in the subsequent extraction, increasing the extraction yield and reducing the environmental impact, pollution and overall cost of the process (Blanco-Llamero et al.). This study demonstrates the promising use of CLEAS as support-free enzymatic biocatalysts for future industrial applications within the current Research Topic.

The studies of Teymennet-Ramírez et al. published in our Research Topic presented Yeast Surface Display System as Strategies for Improvement and Biotechnological Applications. In this context, Yeast surface display (YSD) is defined as a “whole-cell” platform used for the heterologous expression of proteins immobilized on the cell surface of yeast (Teymennet-Ramírez et al.). YSD combines the advantages of eukaryotic systems, such as post-translational changes, correct protein folding and glycosylation, ease of cell culture and genetic manipulation, and allows for protein immobilization and recovery. These systems are advantageous because the proteins displayed on the surface of yeast cells can show more excellent stability against changes in temperature, pH, organic solvents, and proteases. This platform has been used to study protein-protein interactions, antibody design, and protein engineering. Applications for YSD include library screening, complete proteome studies, bioremediation, vaccine and antibiotic development, biosensor production, ethanol production, and Biocatalysis (Teymennet-Ramírez et al.). Therefore, YSD is a promising technology, that is, not yet optimized for biotechnology applications. In this way, knowing the current strategies to improve the efficiency and selection of displayed proteins. In addition, YSD is presented as cutting-edge technology for the vector expression of proteins and peptides. Finally, recent biotechnological applications were summarized by the authors. The different approaches described in the work allowed a better understanding of the strategy to increase protein/peptide interaction and production (Teymennet-Ramírez et al.).

Studies involving computational analysis are present in our topic and research. The purpose of the work published by the authors Azevedo et al. was to carry out a computational study of the biotransformation of α-tocopherol into tocopherol esters, observing the tunnels present in the enzymatic structures as well as the energies that correspond to the transport of the molecules (Azevedo et al.). The computational screening was carried out in work. It was verified that the number of existing tunnels in the enzymatic structure does not presuppose a better biocatalytic result since the lipase with the most significant number of tunnels submitted to in silico analysis in work was the LPP with 39 tunnels, and no other significant results were achieved (Azevedo et al.). However, according to the authors (Azevedo et al.), the lipase that best obtained the results was lipase B from *Candida Antarctica*—CALB (with six tunnels), since the characteristics of the tunnels present in its structure, compared to the ligands submitted to analysis, were more favorable to transport ligands, since that the network tunnel for substrates was distinct from the output tunnel for the product (α-tocopheryl acetate). In addition, compared to the results described in the literature using CALB as a biocatalyst to obtain α-tocopherol acetate, this was reinforced, and the other biocatalysts that were used (LBC and CSF) did not obtain promising results for the authors of the study. Thus, the in silico analysis of biocatalytic reactions in the study performed showed significant results in analyzes before *in situ* reactions. The authors also point out that when performing the in silico analysis, it demonstrates optimization of the execution time in bench experiments since it performs a prediction of the submitted biocatalytic behavior.

The authors synthesized cross-linked (magnetic) enzymatic aggregates with laccase, cellulase, β-galactosidase, and transglutaminase (Hojnik Podrepšek et al.). This study proved the versatility and path used to prepare biocatalysts via CLEAs and mCLEAs. The authors found that the ideal precipitation reagents to obtain the highest residual activity of the resuspended enzyme in the synthesis of CLEAs and mCLEAs were ethanol for cellulase CLEAs, 1-propanol for β-gal and laccase CLEAs, and 2-propanol for TGM CLEAs, with 84%, 102%, 65% and 231% residual activity, respectively (Hojnik Podrepšek et al.). According to the study, the highest relative activities, 118%, and 103%, were obtained after optimizing various process conditions in β-gal CLEAs and β-gal mCLEAs, respectively. Another exciting result presented by the authors was Cellulases CLEAs and TGM mCLEAs that expressed 94% and 73% of relative activity, respectively. The stability of CLEAs and mCLEAs in scCO2 proved useful for enzymatic catalysis in scCO2, where TGM CLEAs and mCLEAs laccase achieved 123% and 105% hyperactivation after exposure to scCO2 conditions at 10 MPa, compared to CLEAs and mCLEAs not exposed. As investigated, the entire study was directed towards preparing two different immobilization techniques, CLEAs, and mCLEAs, and a comparative analysis of four different enzymes with their relative activity and stability under different conditions was also performed (Hojnik Podrepšek et al.). No common rule about optimal immobilization parameters for all enzymes can be predicted. Overall, this study demonstrates that the immobilization of different enzymes on CLEAs and mCLEAs improves stability and can be used in a promising way in different bioapplications (Hojnik Podrepšek et al.).

In the last work of this Research Topic, the authors Hojnik Podrepšek et al. established a methodology to encapsulate yeast cells that exhibit IM7 in calcium alginate beads. The study demonstrated biomaterial-based affinity chromatography to achieve rapid and cost-effective purification of proteins with greater than 90% purity in a single step. Furthermore, the developed system allowed the multi-enzyme coating complex to produce reusable immobilized cells for efficient cascade biotransformation. The present study demonstrated the great potential of use in the laboratory and the industry to design protein products and biocatalysis.

This Research Topic covers the promising and recent Carrier-Free Immobilized Enzymes Project trends for Biocatalysis and applications. The authors present contributions with Original Research Articles, Mini and Full Reviews, and Communications on related topics in this opportunity. Areas to be covered in the Research Topic include Co-immobilization without a multi-enzyme carrier, Cascade reactions, Multipoint covalent linkage without carrier, Stabilization of enzymes by immobilization without a carrier, Modulation of enzymatic properties by immobilization without carrier, Immobilization without carrier multi-subunit, Nanoparticles, and Characterization of immobilized enzymes without carrier. Here, crucial information is presented so that researchers can improve their studies in the search for Carrier-Free Immobilized Enzymes for Industrial Biocatalysis. We would like to thank all the authors, reviewers, and the Editorial Board members for their considerable contributions to supporting the implementation of this special Research Topic.
